# Haematological Features and Urologic Pathologies of Diabetic Subjects at Bafoussam Regional Hospital: A Cross-Sectional Study

**DOI:** 10.1155/2020/6161785

**Published:** 2020-05-28

**Authors:** Arsene T. Signing, Wiliane J. T. Marbou, Veronique P. Beng, Victor Kuete

**Affiliations:** ^1^Department of Biochemistry, University of Dschang, P.O. Box 67, Dschang, Cameroon; ^2^Department of Biochemistry, University of Yaoundé 1, Cameroun P.O. Box 812, Yaoundé, Cameroon

## Abstract

**Background:**

Diabetes mellitus is at the origin of long-term complications.

**Objective:**

This study is aimed at assessing the haematological features and urologic pathologies of diabetic individuals at Bafoussam Regional Hospital.

**Methods:**

This was a cross-sectional study conducted from August 2018 to May 2019 in Bafoussam Regional Hospital, West Cameroon. A structured questionnaire was used to gather sociodemographic data. A trained nurse measured the physical and clinical features. Fasting plasma glucose was determined using the glucose meter Accu-Chek Active system. The full blood count (FBC) was carried out using Automatic full Blood Counter, and the CD4, CD3, and CD8 T-cell counts were determined using the flow cytometry method.

**Results:**

There were 455 diabetic patients, and 50 nondiabetic patients were included. The mean age of diabetic patients (56.94 ± 14.33 years) was higher compared to that of nondiabetic individuals (34.76 ± 14.35 years) (*p* < 0.001). There was a significant relationship between married individuals (*χ*^2^ = 79.19, *p* < 0.001, and *df* = 4), housewife and retired (*χ*^2^ = 1117.38, *p* < 0.001, and *df* = 37), old age (40 years and above) (*χ*^2^ = 79.11, *p* < 0.001, and *df* = 3), and diabetes status. Diabetic patients had an odds of 5.52 to experience a urinary urge as compared to the controls (*p* < 0.001, 95% CI = 2.15-14.22). The majority of haematological parameters were negatively but not significantly correlated with diabetes. Binary logistic regression shows that MCV (*r* = −0.251, OR = 0.778, and 95% CI = 0.617–0.983; *p* = 0.035) and RDW-CV (*r* = −0.477, OR = 0.620, and 95% CI = 0.454–0.848; *p* = 0.003) negatively influence the probability of having diabetes. RDW-SD (*r* = 0.135, OR = 1.144, and 95% CI = 1.014–1.291; *p* = 0.029) positively influences the probability of having diabetes.

**Conclusion:**

This study revealed a significant haematological and urological profile difference according to diabetes status. Research and interventions targeted at diabetic population could help close gaps in diabetes complications.

## 1. Introduction

Diabetes is a disease that is having a significant repercussion on the socioeconomic, physical, and psychological aspects of the lives of the victims. It is a pathology characterized by an abnormal rise of the level of glucose in the blood, which is defined by a fasting glycaemia greater than or equal to 126 mg/dL (measured twice) or glycaemia greater than 200 mg/dL after a meal [1]. It has existed since antiquity but is diagnosed by polyuria alluding to abnormal diuresis in 24 hours [2]. As early as 1797, with the Englishman John Rollo, the first metabolic theories aimed at explaining diabetes were born. According to this author, excess sugar in the urine comes from an abnormal transformation of food carbohydrates by the stomach. In 1848, Claude Bernard demonstrated the glycogenic function of the liver, and it is due to the works of Oskar Minkowski and Joseph Von Mehring that the role of the pancreas was discovered in 1886 at the University of Strasbourg [2]. The removal of the pancreas (or pancreatectomy) in dogs is followed by diabetes, this diabetes being corrected by the pancreas transplant [2].

The world population of diabetes was 360 million in 2000, and it was projected that this rate would increase to 552 million in 2030 [3]. However, new data on the global prevalence of diabetes are thrilling because recent estimates from the International Diabetes Federation indicated that 8.3% of adults (20-79 years) or 382 million people worldwide have diabetes and that number could exceed 592 million in less than 25 years [4]. Some epidemiologists predicted that the economic impact of diabetes and the number of deaths will be more than the one caused by HIV/AIDS. The prevalence of diabetes in Africa and Cameroon is 5.8% and 5% to 6%, respectively [5]. These figures make diabetes to be a public health problem, hence the need to assess all the parameters contributing to the increase in complications linked to this disease. However, the complications of this condition are not the least [4].

Indeed, diabetes is at the origin of long-term complications which can be the source of serious handicaps considerably altering the quality of life [6]. The chronic complications of diabetes include peripheral neuropathy and autonomic neuropathy, retinopathy, diabetic foot, and diabetic kidney disease [6]. The degenerative complications secondary to diabetes, having as main causes of hyperglycaemia and hyperinsulinemia, affect several organs and systems such as the cardiovascular system, eyes, nerves, limbs, and kidneys. Over time, uncontrolled accumulated blood glucose levels lead to tissue damage that can cause urologic complications in diabetic individuals. Diabetes is, therefore, an important risk factor for cardiovascular disease and one of the leading causes of blindness, kidney failure, and amputation for nontraumatic causes in the world [6]. Also, diabetes is a pathology that affects cells of the immune system and makes patients vulnerable to many infections [7]. Along the same lines, studies have shown that diabetes will increase the risk of developing infectious diseases [8]. To provide data regarding the variation of blood cell levels in diabetics, the objective of this work was to assess the distribution of the urologic pathologies and haematological characteristics of diabetes among individuals in the diabetology unit of Bafoussam Regional Hospital.

## 2. Materials and Methods

### 2.1. Study Setting

The study was conducted in Bafoussam Regional Hospital, West Cameroon. The hospital serves as a referral centre for 20 hospitals in western region districts. According to the World Population Review (2020), the total population of Bafoussam is 290768 [9].

### 2.2. Study Design, Period, and Sample Size

A cross-sectional study was conducted from August 01, 2018, to May 29, 2019. The source population was diabetes-confirmed individuals at the diabetology unit, and the nondiabetic individuals came to consult at the diabetes unit of Bafoussam Regional Hospital. The sample size was calculated using the single population proportion formula by considering the sample proportion as 5.8% prevalence of diabetes [5], 0.03 desired precision, 95% confidence interval (CI), and a design effect of 2. Thus, the minimum sample size (*n*) calculated was found to be 468. We, therefore, obtained 455 diabetic patients (41 type I diabetes and 414 type II diabetes) and 50 nondiabetic patients for a total of 505 participants. Type I diabetes and type II diabetes were considered inclusion criteria.

### 2.3. Ethics Considerations

Ethical clearance for this study was obtained from the Cameroon National Research Ethics Committee. We obtained a research certificate from the University of Dschang as well as a research authorization from Bafoussam Regional Hospital. All the participants were duly informed of the study goals, procedures, potential harm and benefits, and cost, as well as the finality of the study. Each patient signed an informed consent form, thereby agreeing to participate in the study. Subsequently, a questionnaire was submitted to them and the collection of samples was carried out following the scientific and ethical standards. All results were coded and kept confidential.

### 2.4. Exclusion Criteria

Pregnant women, tuberculosis patients, and HIV-positive patients were excluded from this study to avoid the possible impact on the anthropometric and laboratory parameters.

### 2.5. Demographic Data Collection

Data on demographics were collected by trained personnel through a face-to-face interview using a structured questionnaire. The study team was composed of laboratory technicians, nurses, and supervisors. Each participant was questioned for age, sex, profession, educational status, and marital status.

### 2.6. Physical Measurements and Clinical Features

Participants' height and weight were measured to calculate the body mass index (BMI). Clinical features were screened by a trained nurse. These clinical features include pollakiuria, dysuria, fever, burn, urinary leakage, and urge to urinate. BMI ≥ 30 kg/m^2^ was considered obese [10].

### 2.7. Biochemical Measurements

Plasma glucose (after an overnight fasting ≥ 8 h) was determined using the glucose meter Accu-Chek Active system [11]. Fasting capillary blood samples were collected three times (for three consecutive hours) from a single study participant, and glucose measurement was carried out within fractions of seconds after sample collection. Then, their average was taken for analysis. The American Diabetes Association diabetes mellitus classification criteria were used for the diagnosis of diabetes. The diagnosis of DM was based on the American Diabetes Association diabetes mellitus classification criteria with fasting blood glucose of ≥126 mg/dL being considered positive for diabetes and fasting blood glucose (FPG) of less than 61 mg/dL to <110 mg/dL being considered normoglycaemic [1]. An FPG level > 126 mg/dL or a casual plasma glucose > 200 mg/dL meets the threshold for the diagnosis of diabetes.

Furthermore, fasting venous blood was collected from each participant, using EDTA tubes for the full blood count and CD4 T-cell count. The full blood count (FBC) was carried out using Automatic full Blood Counter (*Automatic full Blood Counter ERMA*, Japan). The CD4 T-cell count of all the participants were determined using flow cytometry applied in clinical immunology by Becton Dickinson's FACSCount method [12].

### 2.8. Data Analysis

To examine the association between diabetes status with patients' demographic and clinical parameters, we used the chi-squared test for categorical variables and the *t*-test for continuous variables. Binary logistic regression analysis was used to assess the relation between haematological features of study participants versus their diabetes status. *p* values < 0.05 were considered to be significant. All statistical analyses were carried out using SPSS 18.0 (release: July 30, 2009; USA) for Windows (IBM).

## 3. Results

### 3.1. Descriptive Statistics

Out of a sample of 505 research participants, 57.4% (*n* = 290) were females while 42.6% (*n* = 215) were males. With regard to the age range, 224 participants fell within the age group of 41–60 years with a percentage score of 44.4%. These were followed by those within the age range of 61–90 years with 38.0% and frequency of 192. Only 13 participants fell within the age range of 0–20 years with a percentage score of 2.6% (*n* = 13). The majority of participants (*n* = 403) were married with a percentage score of 79.8%, while the least (*n* = 1) was still cohabiting (0.2%). Majority of participants were housewives with 25.0% (*n* = 126), followed by retired civil servants with 17.0% (*n* = 86). The least among them were photographers, majors, and gardener with a percentage of 0.2% each. For the educational level, the majority of those with diabetes had CEP (first school-leaving certificate) (22.2%). Participants that ended their educational level at primary school and have no diploma followed those with CEP with 19.3% (*n* = 88). The least within the sample was a Ph.D. holder with a percentage score of 0.2 (*n* = 1) (Table 1).

### 3.2. Sociodemographic Characteristics of Study Participants according to the Diabetes Status

Sociodemographic features according to diabetes status are presented in Table 2. There were 455 diabetic patients (57.80% females, *n* = 263 and 42.20% males, *n* = 192) and 50 nondiabetic individuals (54% females, *n* = 27 and 46% males, *n* = 23) were included. The mean age of diabetic patients (56.94 ± 14.33 years) was higher compared to that of nondiabetic individuals (34.76 ± 14.35 years) (*p* < 0.001).

At the 0.05 significance level, there was no significant difference between gender and diabetes status within the study sample (*χ*^2^ = 0.27, *p* = 0.606). Despite the differences between males and females for diabetic patients, the control of gender difference was not significant.

There was a significant (*χ*^2^ = 79.19, *p* < 0.001, and *df* = 4) relationship between married individuals and diabetes status. Among the diabetic patients, the majority (83.7%) were those who reported to having been married. These were followed by widows or widowers with a percentage score of 8.8%. At 0.05 significance level, there was a significant difference between profession of respondents and their diabetes status (*χ*^2^ = 1117.38, *p* < 0.001, and *df* = 37).

The educational level showed significant differences with diabetes status at 0.05 significance level (*χ*^2^ = 32.93, *p* < 0.001, and *df* = 10). The majority of those with diabetes had CEP (22.2%). Participants that ended their educational level at primary school and have no diploma followed those with CEP with 19.3% (*n* = 88).

The age range also differed significantly with diabetes status (*χ*^2^ = 79.11, *p* < 0.001, and *df* = 3). The majority of those with diabetes came from the age group 41–60 with a percentage score of 89.7%. In fact, the Spearman correlation analysis indicated a positive significant correlation between age and diabetes (*r* = +0.96, *p* < 0.001).

### 3.3. Clinical Features of the Diabetic and Nondiabetic Study Subjects

Table 3 presents the clinical features of the diabetic and nondiabetic study subjects. All the clinical parameters were significantly associated with diabetes status except for the development of fever (*χ*^2^ = 0.33, *p* = 0.362) and urinary leakage (*χ*^2^ = 1.436, *p* = 0.195). From Table 3, a majority of diabetic patients within the study population reported having had dysuria (*χ*^2^ = 5.73, *p* = 0.021, and OR = 2.39 within a 95% CI of 1.149-4.980). For the urge to urinate for example, diabetic patients had an odds of 5.52 to experience urinary urge as compared to the controls (*p* < 0.001, 95% CI = 2.15-14.22). Also, diabetic patients had an odds of 5.64 to develop obesity as compared to the controls (*p* < 0.001, 95% CI = 1.87-17.03).

### 3.4. *t*-Test Analysis to Compute the Mean Differences in Haematological Parameters between the Diabetic and Control Groups in the Study Sample

A two-independent sample *t*-test was run to compute the mean differences in haematological parameters between the diabetic and control groups in the study sample. Equal variances were assumed in this analysis as the differences between the haematological mean values were close enough (±1.2).

Out of the 20 haematological parameters analysed, the CD4 T-cell blood level (*p* = 0.024, 95% CI = −151.208 to -10.053), % lymphocytes (*p* = 0.024, 95% CI = −6.35 to -0.45), % monocytes (*p* = 0.038, 95% CI = −1.53 to -0.043), GR (*p* < 0.001, 95% CI = −1.72 to -0.54), HCT (*p* = 0.002, 95% CI = −4.862-1.119), RDW-CV (*p* = 0.005, 95% CI = −1.87 to -0.32), and RDW-SD (*p* = 0.003, 95% CI = −8.07 to -1.73) were significantly lower in diabetic patients compared to nondiabetic individuals. The % granulocytes (*p* = 0.002, 95% CI = −6.51 to -0.29) and MCHC (*p* = 0.045, 95% CI = 0.01 to 0.87) were significantly higher in diabetic patients compared to nondiabetic individuals (Table 4). These results reveal haematological disturbance of diabetic patients compared to nondiabetics although the difference in results is not statistically significant for the other parameters.

### 3.5. Relationship between Diabetes Status and Haematologic Parameters

Table 5 presents the correlation between haematologic parameters and diabetes status. The majority of haematologic parameters were negatively but not significantly correlated with diabetes. Binary logistic regression shows that MCV (*r* = −0.251, OR = 0.778, and 95% CI = 0.617–0.983; *p* = 0.035) and RDW-CV (*r* = −0.477, OR = 0.620, and 95% CI = 0.454–0.848; *p* = 0.003) negatively influence the probability of having diabetes. RDW-SD (*r* = 0.135, OR = 1.144, and 95% CI = 1.014–1.291; *p* = 0.029) positively influences the probability of having diabetes.

## 4. Discussion

Diabetes is the most well-known chronic metabolic disease. It is a metabolic disorder characterized by the presence of hyperglycaemia attributable to a reduction in insulin secretion or insulin action or both. Diabetes is a common condition affecting both the young and the elderly, which can lead to acute accidents of various aetiologies mainly metabolic, neurological, cardiovascular, and haematological. This study is aimed at assessing the distribution of the clinical and haematological characteristics of diabetes among individuals in the diabetology unit of Bafoussam Regional Hospital.

This study revealed that out of 505 research participants, 57.4% (*n* = 290) were females while 42.6% (*n* = 215) were males. This result can be explained by a sedentary lifestyle which is notably more accentuated in female individuals compared to males as reported by a previous study [13]. A significant relationship was observed between married individuals and diabetes status. A previous study reported that poor marital quality is associated with many different indicators of poor health such as diabetes [14]. The relationship between marital status and glycaemic control, in particular, the effect of marriage on the onset of diabetes, could probably be due to the pathophysiological and therapeutic characteristics of the disease [15]. Concerning the profession and the diabetes status, a significant difference was observed concerning housewives and retired individuals. The majority of diabetic patients were housewives, followed by retired public servants. This can be explained by the fact that these two categories of the population have a lifestyle of their own, generally characterized by a sedentary lifestyle, an unbalanced diet, travel by motorbike or vehicle even over short distances, and lack of physical activity [16]. A study from Jeddah, Saudi Arabia, indicated that nonsmoker housewives are considered the high-risk group of developing obesity among diabetic patients [17]. Regarding the level of education, the low intellectual level of our study population will justify their lack of knowledge about diabetes and its prevention methods. This may explain the fact that for our study, the majority of diabetics had a first school-leaving certificate followed by participants who completed their education level in primary school and without a diploma. This result is inconsistent with that of Agardh and colleagues in Sweden who demonstrated a considerable burden of diabetes mellitus attributed to lower educational levels in Sweden [18].

This study indicated that the majority of diabetic individuals belonged to the age group between 41 and 60 years. In fact, the Pearson correlation analysis indicated a significant positive correlation between age and diabetes. This is similar to the results obtained in other regions of sub-Saharan Africa and other developing countries such as Ghana [19]. In developed countries like the USA, the correlation was most in the patients who had over the age of 60 years [20]. This can be explained by the fact that it is the category of the socially active population that is easily exposed to certain environmental factors such as the use of alcohol and tobacco, which are the risk factor of diabetes.

About the clinical characteristics of the diabetic and nondiabetic subjects, all clinical parameters were significantly associated with diabetes, except the development of fever and urinary leakage. Diabetic patients in the study population reported having dysuria, characteristic of their type of diabetes. Diabetes status explains the fact that diabetic patients had an odds of 5.52 of experiencing a urinary urge compared to controls and also had an odds of 5.64 of developing obesity compared to controls. Diabetes and urologic pathologies are very common health problems [21]. A recent study has shown that diabetes mellitus independently increases the risk of urinary incontinence in women [22]. Urologic pathologies due to diabetes are a serious kidney-related complication of type 1 diabetes and type 2 diabetes. It affects individuals' kidneys' ability to do their usual work of removing waste products and extra fluid from your body [23].

Analysis of the means of the different haematological parameters between the diabetic group and the control group in the study sample shows that CD4 T-cell blood level, % lymphocytes, % monocytes, GR, HCT, RDW-CV, and RDW-SD were significantly lower in diabetic patients compared to nondiabetic individuals while % granulocytes and MCHC were significantly higher in diabetic patients compared to nondiabetic individuals. These results reveal the lack of immune status of diabetics compared to nondiabetics although the difference in results is not statistically significant for the other parameters. This could be explained by the difference in sample size in the two populations since a very limited number of nondiabetic patients were obtained in the context of our study. Leukocytes represent an important part of immunocompetent cells which are used for defence against infectious agents. Geerlings and Hoepelman noted a decrease in the function of polynuclear cells and monocytes/macrophages in diabetics compared to nondiabetics [24]. These results corroborate those of our study because diabetic patients had granulopenia (drop in the granulocyte level) compared to nondiabetic patients.

The correlation between the haematological parameters and the diabetes status shows that the majority of haematologic parameters were negatively but not significantly correlated with diabetes. MCV and RDW-CV negatively influence the probability of having diabetes while RDW-SD positively influences the probability of having diabetes. This is explained by the fact that diabetes affects haematological cells and functions [25].

These findings are of huge public health prominence since it helped to access the haematological features and urologic pathologies among diabetic individuals compared to nondiabetics. The small size of nondiabetic individuals constitutes one limitation of this study. The major limitation of this study is that diabetes mellitus was diagnosed using a glucose meter from capillary blood; this is not as accurate and reliable as plasma glucose estimation diagnosed using a spectrophotometer/colorimeter.

## 5. Conclusion

This study suggested that the mean of % lymphocytes, % monocytes, % granulocytes, GR, MCHC, RDW-CV, and RDW-SD was significantly different between the diabetic and nondiabetic patients. It also shows that the majority of haematologic parameters were negatively but not significantly correlated with diabetes. MCV and RDW-CV negatively influence the probability of having diabetes while RDW-SD positively influences the probability of having diabetes. This study also suggests that research and interventions targeted at diabetic population could help close gaps in diabetes complications.

## Figures and Tables

**Table 1:** Sociodemographic characteristics of total study participants.

Sociodemographic parameters	Characteristics	Frequency (*n* = 505)	Percentage (%)
Sex	Male	215	42.57
	Female	290	57.43

Matrimonial status	Married	403	79.80
	Widow/widower	66	13.07
	Divorced	7	1.39
	Single	28	5.54
	In a relationship	1	0.20

Profession	Retired	86	17.03
	Teacher	39	7.72
	Housewife	126	24.95
	Trader	45	8.91
	Cultivator	69	13.66
	Cashier	6	1.19
	Cook	6	1.19
	Policeman	4	0.79
	Dressmaker	14	2.77
	Metalworker	2	0.40
	Nurse	12	2.38
	Resourceful	5	0.99
	Carpenter	2	0.40
	Planter	14	2.77
	Potter	2	0.40
	Engineer	3	0.59
	Driver	10	1.98
	Student	9	1.78
	Photographer	1	0.20
	Electrician	3	0.59
	Builder	3	0.59
	Mechanic	7	1.39
	Tailor	2	0.40
	Civil servant	3	0.59
	Mayor	1	0.20
	Gardener	1	0.20
	Municipal agent	3	0.59
	Hairdresser	4	0.79
	Secretary	5	0.99
	Accounting	3	0.59
	Pastor	3	0.59
	Counter	3	0.59
	Stylist	2	0.40

Educational level	GCE O-level	60	11.88
	GCE A-level	54	10.69
	First school-leaving certificate	104	20.59
	HND	8	1.58
	No study conducted	50	9.90
	End of primary studies without a diploma	107	21.19
	Stop high school without diplomas	59	11.68
	Bachelor	35	6.93
	Master	8	1.58
	PhD	1	0.20
	Probatory	19	3.76

Age groups	0-20	13	2.57
	21-40	76	15.05
	40-61	224	44.36
	61-90	192	38.02

GCE: General Certificate of Education; HND: Higher National Diploma.

**Table 2:** Features of participants according to diabetes status.

Sociodemographic parameters	Characteristics	Control patients (*n* = 50)	Diabetic patients (*n* = 455)	*χ* ^2^	*p* value
Sex	Male	23 (46)	192 (42.20)	0.26	0.606
	Female	27 (54)	263 (57.80)		

Matrimonial status	Married	22 (44)	381 (83.73)	79.19	*p* < 0.001
	Widow/widower	26 (52)	40 (8.80)		
	Divorced	2 (4)	5 (1.10)		
	Single	0 (0)	28 (6.15)		
	In a relationship	0 (0)	1 (0.22)		

Profession	Retired	3 (6)	83 (18.24)	117.37	*p* < 0.001
	Teacher	8 (16)	31 (6.81)		
	Housewife	10 (20)	116 (25.50)		
	Trader	3 (6)	42 (9.23)		
	Cultivator	4 (8)	65 (14.29)		
	Cashier	5 (10)	1 (0.22)		
	Cook	3 (6)	3 (0.66)		
	Policeman	3 (6)	1 (0.22)		
	Dressmaker	7 (14)	7 (1.54)		
	Metalworker	1 (2)	1 (0.22)		
	Nurse	2 (4)	10 (2.20)		
	Resourceful	1 (2)	4 (0.88)		
	Carpenter	0 (0)	2 (0.44)		
	Planter	0 (0)	14 (3.08)		
	Potter	0 (0)	2 (0.44)		
	Engineer	0 (0)	3 (0.66)		
	Driver	0 (0)	10 (2.20)		
	Student	0 (0)	9 (1.20)		
	Photographer	0 (0)	1 (0.22)		
	Electrician	0 (0)	3 (0.66)		
	Mason	0 (0)	3 (0.66)		
	Mechanic	0 (0)	7 (1.54)		
	Tailor	0 (0)	2 (0.44)		
	Civil servant	0 (0)	3 (0.66)		
	Mayor	0 (0)	1 (0.22)		
	Gardener	0 (0)	1 (0.22)		
	Municipal agent	0 (0)	3 (0.66)		
	Hairdresser	0 (0)	4 (0.88)		
	Secretary	0 (0)	5 (1.10)		
	Accounting	0 (0)	3 (0.66)		
	Pastor	0 (0)	3 (0.66)		
	Counter	0 (0)	3 (0.66)		
	Stylist	0 (0)	2 (0.44)		

Educational level	GCE O-level	1 (2)	59 (12.96)	32.92	*p* < 0.001
	GCE A-level	3 (6)	51 (11.20)		
	First school-leaving certificate	3 (6)	101 (22.20)		
	HND	2 (4)	6 (1.31)		
	No study conducted	11 (22)	39 (8.57)		
	End of primary studies without a diploma	19 (38)	88 (19.34)		
	Stop high school without diplomas	8 (16)	51 (11.20)		
	Bachelor	2 (4)	33 (7.25)		
	Master	1 (2)	7 (1.54)		
	PhD	0 (0)	1 (0.22)		
	Probatory	0 (0)	19 (4.17)		

Age groups	0-20	5 (10)	8 (1.75)	79.11	*p* < 0.001
	21-40	26 (52)	50 (10.98)		
	40-61	16 (32)	208 (45.71)		
	61-90	3 (6)	189 (41.53)		

GCE: General Certificate of Education; HND: Higher National Diploma.

**Table 3:** Clinical features of the diabetic and nondiabetic study subjects.

Variable	Characteristic frequency (%)		*χ* ^2^ (*p* value)	OR	CI
	Type of diabetes				
	Type I	Type II			
Diabetes	41 (9.01)	414 (90.99)	NA	NA	NA
Control	0 (00)	0 (00)			

	Pollakiuria				
	Yes	No			
Diabetes	254 (55.82)	201 (44.18)	14.760 (0.001)^∗^	0.070	0.0421–0.288
Control	42 (84)	8 (16)			

	Dysuria				
	Yes	No			
Diabetes	407 (89.45)	48 (10.55)	5.730 (0.021)^∗^	2.390	1.149–4.980
Control	39 (78)	11(22)			

	Fever				
	Yes	No			
Diabetes	420 (92.31)	35 (7.69)	0.330 (0.362)	1.330	0.497–3.575
Control	45 (90)	5 (10)			

	Burn				
	Yes	No			
Diabetes	433 (95.16)	22 (4.84)	6.990 (0.017)^∗^	3.204	1.294–7.931
Control	43 (86)	7 (14)			

	Urinary leakage				
	Yes	No			
Diabetes	427 (93.85)	28 (6.15)	1.430 (0.195)	0.311	0.041–2.34
Control	49 (98)	1 (2)			

	Urge to urinate				
	No	Yes			
Diabetes	254 (55.82)	201(44.18)	24.450 (*p* < 0.001)^∗^	5.520	2.15–14.22
Control	46 (92)	4 (8)			

	Obesity				
	Yes	No			
Diabetes	301(66.15)	154 (33.85)	16.310 (*p* < 0.001)^∗^	5.640	1.87–17.03
Control	47 (90)	3 (10)			

	Hospital attendance				
	Yes	No			
Diabetes	119 (26.15)	336 (73.85)	0.719 (*p* = 0.397)	1.348	0.67–2.70
Control	46 (92)	4 (8)			

OR = odds ratio; CI = confidence interval. ^∗^Significant at 0.05 significance level.

**Table 4:** Mean differences in haematological parameters between the diabetic and control groups.

Haematological parameters	Diabetes/control	Frequency	Mean	Std. deviation	Std. error mean	*t*-test			95% CI	
						*t*	*df*	Sig. (2-tailed)		
									Lower	Upper
CD4 T-cells	Diabetes	455	802.369	230.119	10.788	-2245	503	0.025	-151.208	-10.053
	Control	50	883.000	325.757	460.690					

GB (×103 cells/*μ*L)	Diabetes	455	5.465	1.991	0.093	0.478	503	0.633	-0.432	0.711
	Control	50	5.326	1.568	0.221					

Lymphocytes (×103 cells/*μ*L)	Diabetes	455	2.2116	0.71218	0.033	-1493	503	0.136	-0.371	0.050
	Control	50	2.3720	0.79566	0.112					

Monocytes (×103 cells/*μ*L)	Diabetes	455	0.4570	0.16385	0.008	-1415	503	0.158	-0.084	0.013
	Control	50	0.4922	0.19436	0.027					

Granulocytes (×103 cells/*μ*L)	Diabetes	455	2.807	1.694	0.079	1190	503	0.235	-0.191	0.777
	Control	50	2.514	1.230	0.174					

% lymphocytes	Diabetes	455	41.740	10.027	0.470	-2267	503	0.024	-6.349	-0.453
	Control	50	45.142	10.466	1.480					

% monocytes	Diabetes	455	8.852	2.511	0.117	-2079	503	0.038	-1.527	-0.043
	Control	50	9.638	2.745	0.388					

% granulocytes	Diabetes	455	49.3925	10.904	0.511	3047	503	0.002	1.762	8.162
	Control	50	44.430	11.176	15.805					

Haemoglobin	Diabetes	455	12.254	1.800	0.084	-1465	503	0.144	-0.947	0.138
	Control	50	12.659	2.293	0.324					

GR	Diabetes	455	4.762	0.628	0.029	-3743	503	0.000	-1.717	-0.535
	Control	50	5.888	6.181	0.874					

HCT	Diabetes	455	42.166	6.137	0.287	-3140	503	0.002	-4.862	-1.119
	Control	50	45.158	8.412	1.189					

MCV	Diabetes	455	88.457	6.508	0.305	1560	503	0.119	-0.430	3.753
	Control	50	86.796	11.475	1.622					

MCH	Diabetes	455	25.725	1.985	0.093	0.497	503	0.620	-0.443	0.742
	Control	50	25.576	2.366	0.334					

MCHC	Diabetes	455	29.007	1.336	0.062	2006	503	0.045	0.009	0.874
	Control	50	28.566	2.424	0.342					

RDW-CV	Diabetes	455	14.203	2.321	0.108	-2790	503	0.005	-1.872	-0.324
	Control	50	15.302	4.674	0.661					

RDW-SD	Diabetes	455	46.101	5.838	0.273	-3035	503	0.003	-8.066	-1.727
	Control	50	50.998	29.792	4.213					

PLT	Diabetes	455	238.079	77.592	3.637	0.867	503	0.386	-12.761	32.939
	Control	50	227.990	82.293	11.638					

MPV	Diabetes	455	9.981	0.77383	0.036	1307	503	0.192	-0.077	0.384
	Control	50	9.828	0.92097	0.130					

PDW	Diabetes	455	15.000	6.537	0.306	0.692	503	0.489	-1.184	2.471
	Control	50	14.356	2.056	0.290					

PCT (%)	Diabetes	455	0.232	0.069	0.003	0.703	503	0.482	-0.013	0.027
	Control	50	0.225	0.074	0.010					

CD: cluster of differentiation; FBC: full blood count; GB: white blood cells; GR: red blood cells; HCT: haematocrit; HIV: human immunodeficiency viruses; MCV: mean corpuscular volume; MCH: mean corpuscular haemoglobin; MCHC: mean corpuscular haemoglobin concentration; MPV: mean platelet volume; RDW-CV: red blood cell distribution width-coefficient of variation; RDW-SD: red blood cell distribution width-standard deviation; PCT: procalcitonin; PDW: platelet distribution width; PLT: platelet. ^∗^Significant at 0.05 significance level.

**Table 5:** Correlation of haematologic parameters and diabetes status.

Parameters	*r*	S.E.	Wald	*df*	Sig.	OR	95% CI	
							Lower	Upper
CD4 T-cells	-0.001	0.001	3.072	1	0.080	0.999	0.997	1.000
GB (×103 cells/*μ*L)	3.317	3.259	1.036	1	0.309	27.583	0.046	16379.923
Lymphocytes (×103 cells/*μ*L)	-2.726	3.105	0.771	1	0.380	0.065	0.000	28.769
Monocytes (×103 cells/*μ*L)	-5.020	5.375	0.872	1	0.350	0.007	0.000	248.436
Granulocytes (×103 cells/*μ*L)	-3.662	3.254	1.266	1	0.260	0.026	0.000	15.119
% lymphocytes	0.190	0.184	1.066	1	0.302	1.209	0.843	1.735
% monocytes	0.382	0.280	1.852	1	0.174	1.465	0.845	2.538
% granulocytes	0.290	0.193	2.248	1	0.134	1.336	0.915	1.951
Haemoglobin	0.581	0.910	0.407	1	0.523	1.787	0.300	10.635
GR	-0.169	0.821	0.043	1	0.837	0.844	0.169	4.220
HCT	-0.267	0.281	0.906	1	0.341	0.765	0.441	1.328
MCV	-0.251	0.119	4.439	1	0.035	0.778	0.617	0.983
MCH	0.801	0.440	3.313	1	0.069	2.228	0.940	5.277
MCHC	-0.494	0.708	0.488	1	0.485	0.610	0.152	2.442
RDW-CV	-0.477	0.159	8.985	1	0.003	0.620	0.454	0.848
RDW-SD	0.135	0.062	4.770	1	0.029	1.144	1.014	1.291
PLT	0.019	0.024	0.626	1	0.429	1.019	0.973	1.067
MPV	0.643	0.743	0.749	1	0.387	1.903	0.443	8.166
PDW	1.107	0.976	1.287	1	0.257	3.024	0.447	20.465
PCT (%)	-14.314	24.312	0.347	1	0.556	0.000	0.000	3.004*E*14

CD: cluster of differentiation; FBC: full blood count; GB: white blood cells; GR: red blood cells; HCT: haematocrit; HIV: human immunodeficiency viruses; MCV: mean corpuscular volume; MCH: mean corpuscular haemoglobin; MCHC: mean corpuscular haemoglobin concentration; MPV: mean platelet volume; RDW-CV: red blood cell distribution width-coefficient of variation; RDW-SD: red blood cell distribution width-standard deviation; PCT: procalcitonin; PDW: platelet distribution width; PLT: platelet. ^∗^Significant at 0.05 significance level.

## Data Availability

All data generated or analysed during this study are included in this published article and supporting file.

## Supplementary Materials

Supplementary Materials Supplementary Materials (.xlsx): all raw data generated to support the findings of this study.
